# Spliceosome mutations in myelodysplastic syndromes and chronic myelomonocytic leukemia

**DOI:** 10.18632/oncotarget.749

**Published:** 2012-11-30

**Authors:** Virginie Chesnais, Olivier Kosmider, Frederik Damm, Raphael Itzykson, Olivier A. Bernard, Eric Solary, Michaela Fontenay

**Affiliations:** ^1^ Institut Cochin; ^2^ INSERM U1016; ^3^ CNRS UMR8104; ^4^ Université Paris Descartes, Paris France; ^5^ Assistance Publique-Hôpitaux de Paris, Service d'Hématologie Biologique, Hôpitaux universitaires Paris-Centre, Paris, France; ^6^ Institut Gustave Roussy, Villejuif, France; ^7^ INSERM, U985, Villejuif, France; ^8^ INSERM U1009, Villejuif, France; ^9^ Université Paris Sud-11, Faculté de Médecine, Le Kremlin-Bicêtre, France

**Keywords:** splicing, alternative splicing, mutations, myeloid malignancies

## Abstract

The recently discovered spliceosome mutations represent a group of acquired genetic alterations that affect both myeloid and lymphoid malignancies. A substantial proportion of patients with myelodysplastic syndromes (MDS), chronic myelomonocytoic leukemia (CMML) or chronic lymphocytic leukemia (CLL) harbor such mutations, which are often missense in type. Genotype-phenotype correlations have been observed, including the clustering of ring sideroblasts with SF3B1 mutations in MDS. Spliceosome mutations might result in defective small nuclear ribonucleoprotein complexes assembly on the pre-mRNA, deregulated global and alternative mRNA splicing, nuclear-cytoplasm export, and unpliced mRNA degradation, and thus may alter the expression of multiple genes. In the current review, we discuss the potential role of these mutations in cell transformation and how they could impact the therapeutic approaches.

## INTRODUCTION

Most recurrent somatic mutations recently evidenced in myeloid malignancies, including myelodysplastic syndromes (MDS), myeloproliferative neoplasms (MPN), and acute myeloid leukemias (AML), affect genes implicated in the regulation of gene expression (*RUNX1, TEL/ETV6, TP53, TET2, ASXL1, EZH2, IDH1/2, DNMT3A*). In MDS, high throughput sequencing comparing DNA from bone marrow mononuclear cells to germ-line DNA, which identified around 10 acquired mutations per patient sample, suggested that alterations in the control of translation may be implicated in the pathogenesis of myeloid disorders [1, 2, 3]. Impaired protein translation had been identified previously in the erythroid lineage of patients with a 5q- syndrome, a particular MDS subtype characterized by the haplo-insufficiency of *RPS14* gene encoding a ribosomal protein of the small ribosome subunit [4]. Exome sequencing of MDS samples has shown that altered translation could be related also to recurrent mutations in spliceosomal protein genes (*SF3B1, SRSF2, ZRSR2, U2AF35*) whose products control the mechanism of pre-messenger RNA (pre-mRNA) splicing. Similar analyses conducted in chronic lymphocytic leukemia (CLL), where DNA from tumor CD19^+^CD5^+^ lymphocytes and non-tumor cells were compared, also identified mutations affecting the control of splicing mechanisms [5,6] indicating that similar mutations could be observed in both myeloid and lymphoid neoplasms [7, 8]. In addition to myeloid and lymphoid neoplasms, splice gene mutations and amplifications were identified in solid tumors such as breast and lung cancers [9]. Altogether, these observations suggest that aberrations in genes of the spliceosome machinery could contribute to the onset of cancers [10, 11].

### Spliceosome functioning

Gene expression in eukaryotic cells includes the processing of pre-mRNA into mature forms of mRNA through the splicing of introns and ligation of exons in the nucleus [for review, ref. 12]. Most of the human genes are processed to produce two or more transcripts by a mechanism of alternative splicing *ie* the alternative inclusion or exclusion of coding exons, or part of coding exons, in a cell type-specific manner. This mechanism generates a large diversity of mRNA species and is submitted to quality check by a “nuclear surveillance”. When 3' processing/ polyadenylation is inefficient or compromised by gene mutations, then the nuclear exosome is recruited to degrade aberrantly spliced and read-through pre-mRNA [13, 14]. The spliceosomes are made up of multiple and large small nuclear ribonucleoprotein (RNP) complexes that catalyze the splicing reaction. The vast majority of introns (~99%) are spliced by a so-called “U2-dependent spliceosome” [15]. The major U2-dependent spliceosome contains more than 150 proteins and 5 small nuclear (sn) RNAs (U1, U2, U4/U6, and U5). Early steps include the recruitment of snRNP complexes to newly transcribed pre-mRNA for the recognition of 5' and 3' exon/intron junctions and later steps include the interaction between the 5' and 3' complexes to catalyse the excision of introns.

**Figure 1:**
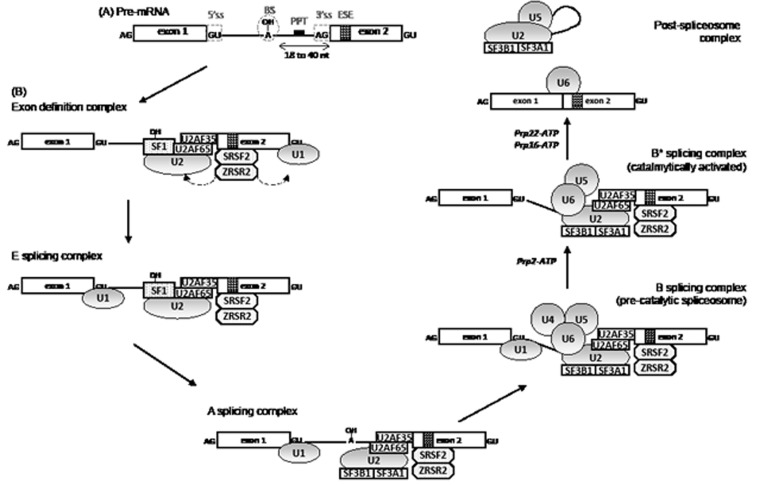
Pre-mRNA splicing mechanism by the U2-type spliceosome (A) A pre-mRNA contains conserved sequences in intron and exon which are necessary for splicing. The 5'splicing site (ss) and the 3' splicing site define the intron and participate in the assembly of the spliceosome together with the branch site (BS) and the polypyrimidine tract (PPT). Within exons, sequence like the exon splicing enhancer (ESE) also participates to the splicing process. (B) RNA splicing is initiated by the recognition of 5' splice site by an U1 snRNP complex. The splicing factor 1 (SF1) binds to the branchpoint sequence located upstream of the 3' end of the intron and a complex which contains an U2 auxiliary factor (AF) 35/65 heterodimer binds respectively to the polypyrimidine tract and the 3'splicing site. This complex is stabilized by ZRSR2 and one protein of the serine-arginine (SR)-rich domain family proteins, SRSF1 or SRSF2. A poorly understood mechanism leads to a switch from an exon-defined to intron-defined splicing complex which forms the E complex. Then, the U2 snRNP complex which contains the SF3b subcomplex, made of SF3B1 and SF3A1 proteins, displaces SF1 to generate the A splicing complex. This step is followed by the integration of the U4/U6-U5 snRNP complexe leading to the formation of the pre-catalytic spliceosome. Lastly, the conformation changes with the release of U1 and U4 snRNP for the spliceosome to become catalytically competent with the help of Prp2, Prp22 and Prp16 DExHD-box helicases. After catalysis, the spliceosome disassembles and is recycled.

RNA splicing is initiated by the recognition of 5' splice site by an U1 snRNP complex. The splicing factor 1 (SF1) complex, *via* its component SF3B1, binds to the branchpoint sequence located upstream of the 3' end of the intron to protect the region before the splicing reaction. A complex that contains an U2 auxiliary factor (AF) 35/65 heterodimer, ZRSR2, and one of the serine-arginine (SR)-rich splicing factors, SRSF1 or SRSF2, is recruited to the polypyrimidine tract located between the branchpoint and the 3' splice site. SRSF1 and SRSF2 play a role in preventing exon skipping, thus regulating alternative splicing. U2AF65 binds the polypyrimidine tract, while U2AF35, also known as U2AF1, interacts with the AG splice acceptor dinucleotide of the target intron at the 3' splice site. ZRSR2 selectively binds to the 3' splice site of U2 dependent pre-mRNA. Together with U2AF heterodimer, SF1 participates to the establishment of the E splicing complex. Then, the U2 snRNP complex, which contains the SF3b subcomplex made of SF3B1 and SF3A1 proteins, displaces SF1 to generate the A splicing complex. This step is followed by the incorporation of the U4/U6-U5 snRNP complexes. Lastly, the release of U1 and U4 snRNP changes the conformation of the complex for the spliceosome to become catalytically competent [16]. The E/A complexes are involved in the recognition of pre-mRNA during the very first steps of splicing, more precisely to the recognition of the polypyrimidine track and the acceptor splice site and also the exon splice enhancer (Figure 1).

Most pre-RNA splicing events occur co-transcriptionally. The recruitment of spliceosome complexes to actively transcribing genes is finely ordered starting with the association of U1 snRNP to a newly formed 5' splice site followed by U2 and U5 snRNP when the intron is fully synthesized. The direct interaction between U1 snRNP and/or splicing regulatory proteins and the DNA polymerase II suggests an interconnection between transcription and mRNA splicing [17]. There is also a link between splicing and mRNA export from the nucleus and translation, which is mediated by SR family proteins. These proteins are recruited to pre-mRNA for splicing in a hyperphosphorylated state and become partially dephosphorylated as the splicing reaction proceeds. They also function as adaptors for spliced mRNA export when in hypophosphorylated state [18]. The SR protein, SF2, also enhances translation initiation through the recruitment of 40S ribosomal S6 kinase 1, which activates translation initiation when activated by the TOR signaling cascade.

Because introns often contain termination codons in frame with the upstream protein coding sequences, a leakage of unspliced pre-mRNA into the cytoplasm could occur. To prevent the production of aberrantly truncated proteins, mRNA species containing premature termination codon are degraded through the nonsense-mediated mRNA decay (NMD), a translation-dependent degradation pathway in the cytoplasm. This pathway is also a key regulator of pre-mRNA splicing factors cell content. When intracellular concentrations become too high, splicing factors regulate their own expression by targeting their mRNAs to NMD. Again, this establishes a link between splicing factors and NMD [19]. In addition, the SF3b subcomplex in the U2 snRNP is involved in the retention of aberrant pre-mRNA in the nucleus. Thus, splicing machinery components participate to the quality check allowing the translation of well-processed mature mRNA in the cytoplasm.

Recent studies have identified striking differences of alternative splicing between embryonic stem cells and differentiated cell populations [20, 21, 22]. For instance, an embryonic stem cell (ESC)-specific alternative splicing event changes the DNA-binding preference of the Forkhead family transcription factor FOXP1, stimulating the expression of transcription factor genes required for the maintenance of pluri-potency, and repressing the expression of genes driving ESC differentiation [22]. Thus, the splicing needs to be tightly regulated to avoid the deregulation of cell differentiation processes.

### Consequences of spliceosome alterations

Most of the splice factors can be mutated in hematological malignancies or solid tumors, some of them being implicated in splicing or in alternative splicing, and also in some extra-splicing functions. Knockdown of these factors alters the splicing pattern of a subset of genes, and changes in their expression level have been linked also to the pathogenesis of cancer [9, 23]. For instance, the embryonic M2 isoform of the pyruvate kinase is re-expressed through alternative splicing in lung cancers, thus promoting the aerobic glycolysis in tumor cells, also known as the Warburg effect. The mechanism of this re-expression involves three heterogeneous nuclear RNP, the polypyrimidine tract binding protein PTB, hnRNPA1 and hnRNPA2, which bind to flanking sequences of exon 9 and repress the exclusion of exon 10 [24]. Expression of the three hnRNPs can be driven by the c-Myc oncogene that upregulates their transcription [25]. During c-Myc-driven cell transformation, the upregulation of splicing factor SRSF1 also results in aberrant alternative splicing events in cell cycle, apoptosis and signalling regulatory genes *BCL2L11, BIN1, MNK2, S6K1, CCND1, RAC1*, and *RON* [26, 27].

Given that splicing is often tightly coupled to transcription [28], alternative splicing might be affected by chromatin structure and histone modification, for instance through the direct recruitment of the splice factor MRG15/MORF4L1 to the H3K36me3 chromatin [29]. Conversely, *SF3B1* interacts with the Polycomb repressive complex (PRC), and the lack of *SF3B1* impairs PRC function, which may influence the chromatin structure and the transcription of *HOX* genes [30]. In addition, mutations affecting the nuclear degradation of non-coding RNA by exosome are likely involved in cell transformation. Inactivating mutations in the exonuclease gene *DIS3*, which encodes the catalytic subunit of the exosome [31] or in the exoribonuclease gene *SEP1/KEM1*, could lead to an accumulation of non-coding RNA species and interfere with transcription [32]. *DIS3* mutations have already been reported in AML [33]. Changes targeting “splice genes” may therefore alter the maturation of pre-mRNA by affecting the spliceosome functions, and also the efficiency of transcription and the degradation of non-coding RNA [14]. This may lead to (i) degradation of unstable mRNA mimicking a loss of tumor suppressor gene or (ii) aberrant alternative splicing increasing the oncogenic potential of the defective proteins with a dominant negative effect. The role of alternative splicing in cell differentiation has been well established, and deregulation of these processes may also be involved in cell transformation [22]. Thus, incidence of splice gene mutations could depend on whether they occur in the hematopoietic stem cell or in more committed progenitors.

### Frequence of splice gene mutations in MDS and other myeloid malignancies

Mutations in *SF3B1* gene were initially identified by whole exome sequencing in 6 cases of 9 MDS, 8 of them being refractory anemia with ring sideroblasts, and a large scale mutational analysis confirmed the high frequency of *SF3B1* mutations in MDS (20%) compared to other myeloid neoplasms (AML 5%; MPN 3%).[3] Yoshida and colleagues analyzed 29 MDS cases and identified multiple mutations of the RNA splicing machinery including *SF3B1, U2AF35, SRSF2, ZRSR2, SF3A1* and *PRPF40B* in 16 cases [1]. Those mutations were frequent in MDS (45-87%), CMML (55%), and secondary AML (26%), but rare in *de novo* AML (7%), MPN (9%), primary myelofibrosis (6.5%), and pediatric myeloid neoplasms including JMML [34-41]. We reported splice gene mutations in 95/221 MDS patients with a frequence of 16, 11, 11 and 5% for *SF3B1, SRSF2, ZRSR2* and *U2AF35* mutations, respectively [34]. In CMML, mutations in *SRSF2* gene, which are frequently associated to mutations in *TET2* gene, are identified in ~50% of the patients whereas mutations in other splice genes are found in an additional 10% of these patients [42-43]. The frequence of *SF3B1* mutations is equivalent in *de novo* and secondary AML while that of *U2AF35* mutations is higher in secondary AML than in *de novo* diseases (Table 1).

**Table 1:** Frequency and impact on prognosis of splicing factor mutations in haematological malignancies including MDS, MPN, CMML, JMML, AML and CLL

Study	Ref	SF3B1	SRSF2	ZRSR2	U2AF35 / U2AF1	Prognosis
**MDS**						
Yoshida et al, 2011	1	75.3% (55/73) RS patients	5,50%	1,40%	0	
	1	6.5% (10/155) non RS patients	11,60%	7,70%	11,60%	
Papaemmanuil et al, 2011	3	20.3% (72/354)	NA	NA	NA	Favorable
Damm et al, 2012a	48	14.7% (47/317)	NA	NA	NA	No
Patnaik et al, 2011	44	49.5% (53/107) RS patients only	NA	NA	NA	Favorable
Malcovati et al, 2011	45	29,4% (162/551)	NA	NA	NA	Favorable
Visconte et al, 2012a	41	68.8% (22/32) RS patients	NA	NA	NA	
	41	0% (0/24) non RS patients	NA	NA	NA	
Thol et al, 2012	35	14,5% (28/193)	12,1% (24/193)	3,1% (6/193)	7,3% (14/193)	Poor
Damm et al, 2012b	34	16,4% (37/221)	11,1% (25/221)	11,1% (25/221)	5,4% (12/221)	Poor
Bejar et al, 2012	36	22% (64/288)	13% (36/288)	NA	16% (46/288)	No
Cui et al, 2012	47	53% (55/104) MDS-RS	NA	NA	NA	Favorable
Jeromin et al, 2012	39	87,2% (41/47) MDS-RS	NA	NA	NA	
Makishima et al, 2012	37	28,4% (25/88, RS and non RS)	10,2% (9/88)	NA	9,0% (8/88)	Poor (SRSF2, U2AF35)
Visconte et al, 2012b	46	39% (37/93), RARS: 68% (13/19)	NA	NA	NA	Favorable
Graubert T et al, 2011	2	NA	NA	NA	8,7% (13/150)	Poor
Qian et al, 2012	52	NA	NA	NA	6,3% (6/96)	No
Wu et al, 2012	53	NA	14,6% (34/233)	NA	NA	Poor
**MPN**						
Yoshida et al, 2011	1	0% (0/53)	1,90%	1,90%	1,90%	
Papaemmanuil et al, 2011	3	ET: 3% (6/189) - PMF: 4% (6/136) - PV: 0/95	NA	NA	NA	
Zhang et al, 2012	38	PMF : 5,3% (2/38)	2,6% (1/38)	5,6% (2/38)	2,6% (1/38)	
Lasho et al, 2012a	49	PMF : 6.5% (10/155)	NA	NA	NA	No
Lasho et al, 2012b	55	PMF : 7% (12/187)	17% (32/187)	NA	NA	Poor
**CMML**						
Yoshida et al, 2011	1	4.5% (4/88)	28,40%	8%	8%	
Papaemmanuil et al, 2011	3	4.7% (5/106)	NA	NA	NA	
Makishima et al, 2012	37	3,0% (2/66)	21,2% (14/66)	NA	7,5% (5/66)	
Visconte et al, 2012a	41	8,3% (5/60)	NA	NA	NA	
Meggendorfer et al, 2012	42	NA	47% (129/275)	NA	NA	No
Malcovati et al, 2011	45	6.5% (4/62)	NA	NA	NA	
Abu Kar et al, 2012	54	6% (5/87)	32% (28/87)	NA	13% (11/87)	Poor
**JMML**						
Hirabayashi et al, 2012	40	0	2/116	NA	0	
Abu Kar et al, 2012	54	0	0	NA	0	
**AML**						
Yoshida et al, 2011	1	4,8% (3/62) sAML	6,5% (4/62)	1,6% (1/62)	9,7% (5/62)	
Yoshida et al, 2011	1	2,6% (7/151) nAML	0,7% (1/151)	0	1,3% (2/151)	
Papaemmanuil et al, 2011	3	5.3% (3/57) nAML	NA	NA	NA	
Makishima et al, 2012	37	3,7% (2/54) sAML	13% (7/54)	NA	9,2% (5/54)	
Makishima et al, 2012	37	7,2% (4/55) nAML	0	NA	10,9% (6/55)	
Zhang et al, 2012	38	3,7% (2/54) nAML	5,6% (3/54)	5,6% (3/54)	1,9% (1/54)	
Zhang et al, 2012	38	8,6% (8/95) sAML	23,7% (22/95)	1,8% (2/95)	5,7% (5/95)	Poor
Visconte et al, 2012a	41	4,7% (2/44) nAML	NA	NA	NA	
Visconte et al, 2012a	41	5,9% (3/50) sAML	NA	NA	NA	
Malcovati et al, 2011	45	5.3% (2/38) sAML	NA	NA	NA	
Qian et al, 2012	52	NA	NA	NA	2,5% (7/275) nAML	
**CLL**						
Wang et al, 2011	5	15% (14/91)	NA	NA	NA	Poor
Quesada et al, 2011	6	9.7% (27/279)	NA	NA	NA	Poor
Rossi et al, 2011	50	7.5% (27/360)	NA	NA	NA	Poor
Oscier et al, 2012	51	17% (84/494)	NA	NA	NA	Poor

This table summarizes the main studies establishing the rate (%) of somatic mutations of SF3B1, SRSF2, ZRSR2 and U2AF35/U2AF1 in haematological malignancies. The number of positives cases among the global cohort is indicated in parenthesis for each study. When available, the impact on the prognosis (poor, favourable or no impact) is indicated in the last column.

For MDS, authors may have distinguished patients with or without Ring Sideroblasts (RS).

In some cases, secondary AML (sAML) and de novo AML (nAML) have been studied separately. NA: Not Available, RS: Ring Sideroblasts, ET: Essential Thrombocytemia, PMF: Primary MyeloFibrosis, PV: Polycythemia Vera, MDS: Myelo Displastic Syndrome, CMML : Chronic Myelo Monocytic Leukemia, MPN: Myelo Proliferative Neoplasms, JMML: Juvenile Myelo Monocytic Leukemia, CLL: Chronic Lymphocytic Leukemia.

*SF3B1* gene is located on 2q33.1, *SRSF2* on 17q25.2, and *U2AF35* on 21q22 chromosomes. With the exception of *ZRSR2*, which is located on the Xp22.2 chromosome, thus can be fully inactivated by heterozygous mutations in males, the other spliceosome mutations are missense mutations, often recurrently targeting a single amino acid. For example, 50% of *SF3B1* mutations target the K700 amino-acid, most of the *SRSF2* mutations are missense mutations or deletions at P95, while *U2AF35* mutations in S34 and Q157 affect the two canonical zinc finger domains. In most cases, splice gene mutations are mutually exclusive. This type of mutational profile often indicates a gain of function that could possibly alter spliceosome functioning. As a result, the expression of many genes could be affected because of intron presence or the omission of exons in the mature transcript, or the deregulation of alternative splicing. *In vitro*, cells transfected with mutant *U2AF35* present with an increase in exon skipping, thus confirming a gain of function, leading to a decrease in cell proliferation capacities and to a lower reconstitution capacity to competitive assay in mice [1,2]. Interestingly, whole mRNA deep sequencing comparing patients with a splice gene mutation to a patient without mutation revealed no genome-wide increase in intron retention, but an alternative splicing pattern in specific genes, including *TET2* and *RUNX1* [37].

### Impact of splice gene mutations on MDS phenotype and clinical outcome

In MDS, mutations in *SF3B1* are associated with lower hemoglobin levels than other splice gene mutations and cluster with the presence of ringed sideroblasts and with *DNMT3A* mutations [1, 3, 34-38, 44-48]. *SF3B1* mutations are strongly predictive of the presence of ringed sideroblasts, whatever the WHO subtype [37, 45]. *Sf3b1*^+/−^ mice also show an excess of ring sideroblasts in the bone marrow, without features of anemia, while the homozygous knockdown of *Sf3b1* is lethal in embryos [30, 46].

Splice gene mutations correlate with distinct clinical phenotypes. For example, mutations in *SRSF2* and *U2AF35* are frequently detected in patients with advanced stages of MDS [34, 35]. *SRSF2* mutated patients exhibit pronounced thrombocytopenias, while *ZRSR2* mutated patients often display isolated neutropenias. The two mutations are more frequently associated with *TET2* mutations than by chance [35]. *SRSF2* mutations are more frequent in CMML (up to ~50%) than in MDS (~10%) [34, 43]. Also, patients with *U2AF35* mutations, who have an increased prevalence of chromosome 20 deletions and *ASXL1* mutations, have more frequently with advanced stages and sAML.

*SF3B1* mutations have been associated with a good impact on overall survival and disease progression to AML in large series of patients, including a majority of refractory anemia with RS (RARS) [3, 44-47]. However, the good prognostic of RARS could induce a bias in these analyses and the subtype-independent prognostic impact of the mutation in MDS is still a matter of debate [34-37, 48]. By contrast, SF3B1 mutations have no impact on primary myelofibrosis and support a poor prognosis on overall survival in CLL [49, 50, 51].

The prognostic impact of *U2AF35* mutations is also a controversial issue in myeloid malignancies [2, 34-37, 52], while *SRSF2* mutations have a negative impact on MDS, MDS/MPN or MPN survival and MDS disease progression [34, 35, 37, 38, 53-55]. This is less clear in CMML in which the prognosis could depend on the combined mutations, *i.e.* the poor prognostic value of the mutations disappears when combined with a mutation in *RUNX1* [43]. Thus, the frequent combination of *SRSF2* mutations with *ASXL1, TET2* and *RUNX1* mutations could blur the picture [43]. For example, a multivariate analysis conducted in a cohort of 221 MDS patients whose mutational status was determined for 16 different genes, an inferior overall survival and a higher AML transformation rate was found for the genotype *ZRSR2*^mut^/*TET2*^wt^ [34] (Table 1).

### Targeting the splice

Several anticancer drugs are spliceosome inhibitors. Heterozygous splice gene mutations mainly result in a gain of function of the protein that has to be inhibited. Given that homozygous inactivation of splice genes is lethal in mice, spliceosome inhibitors have to be more toxic to heterozygous mutant cells than normal ones.

Such a therapeutic avenue could exist for FR901464, a natural bacterial product that was causes cell cycle arrest at G1 and G2/M phases in tumor cells. Recent evidence demonstrates that this compound and its methylated derivative spliceostatin A inhibits pre-mRNA splicing by non convalent binding to the SF3b complex in the U2 snRNP, leading to the leak of unspliced mRNA to be translated in the cytoplasm, and a partial accumulation of mRNA-polyA in the nucleus [56]. The stable derivative of FR901464, known as meayamycin, produces ring sideroblasts in normal erythroid cell cultures [46] suggesting that, if it efficiently reduces tumor cell growth, it may also alter residual normal erythropoiesis.

Another anti-tumor compound is the biflavonoid isogenkgetin, which prevents the stable recruitment of the U4-U6/U5 tri-snRNP to the pre-mRNA, resulting in the accumulation of pre-complex A [57]. Pladienolide is a natural macrolide with antitumor activity that binds the SF3b complex and was shown to inhibit mRNA splicing and cell proliferation in colon cancer cells but a drug resistance mechanism has already be identified, i.e. a mutation at Arg 1074 in the *SF3B1* gene decreases the binding affinity of pladienolide to its target [58]. Additional *in vitro* studies on clonal and normal hematopoiesis and pre-clinical studies are ongoing to determine if these compounds deserve to be tested in MDS.

Some genes can produce both oncogenic and tumor suppressor proteins, based on the inclusion or exclusion of specific exons. In most cancers, alternative splicing of many genes is deregulated. Thus, another therapeutic approach that has been envisioned is the modulation of alternative splicing to prevent the generation of oncogenic forms of some proteins. Such an approach could apply to the alternative splicing of genes encoding pro-angiogenic (VEGF), signalling (STAT3), or pro-apoptotic (Bax) proteins. For example, borrelin-1, which was shown to be a potent inhibitor of angiogenesis, targets a spliceosome-associated protein, FBP21, leading to a modification of the ratio between VEGF isoforms in favour of the anti-angiogenic isoform [59]. The STAT3 transcription factor, which is constitutively activated in a number of human cancers, exists as a full-length STAT3a isofrom and a shorter STAT3b that lacks part of exon 23, due to an alternative splicing event. The short isoform lacks the transactivation domain but still dimerizes with STAT3a and binds to DNA without inducing transcription, thus may act in a dominant-negative manner to induce apoptosis and inhibit tumor growth. An antisense oligonucleotide (AON) targeting the acceptor splice site of STAT3a exon 23 induces a switch from STAT3a to STAT3b in cultured tumor cells and increases cell death, whereas *in vivo* injections of the morpholino counterparts into implanted tumors in mice results in tumor regression [60].

## CONCLUSION AND PERSPECTIVES

Mutations targeting “splice genes” appear to be deleterious to cell growth. Such a detrimental mutation could hitchhike on a previous mutation to become advantageous, a scenario called “epistasis” [61]. An epistatic relation could exist, for example, between *SRSF2* and *TET2* genes, with *SRSF2* mutation being detrimental in the context of a *TET2*-wildtype cell, and advantageous to the *TET2*-mutated clone, thus inducing a *TET2*/*SRSF2* synthetic viability and generating a MDS or a CMML phenotype [34, 62]. According to this scenario, the prognostic significance of *SRSF2* mutations could depend on the genetic background on which the mutation occurs. Very few studies have examined the specific targets of deregulated spliceosome and the mechanisms of action of these mutations, which will require the development of sophisticated models. Extensive clinical studies will also contribute to decipher the respective contribution of mutations in splice genes, epigenetic regulators, signaling molecules, and their combinations, leading to a refined molecular classification of malignancies and hopefully to the development of targeted therapies.
